# Non anti-coagulant factors associated with filter life in continuous renal replacement therapy (CRRT): a systematic review and meta-analysis

**DOI:** 10.1186/s12882-017-0445-5

**Published:** 2017-02-20

**Authors:** Matthew Brain, Elizabeth Winson, Owen Roodenburg, John McNeil

**Affiliations:** 1 School of Public Health and Preventive Medicine, Monash University, Melbourne, VIC Australia; 2 The Alfred Intensive Care Unit, Melbourne, VIC Australia; 3 Launceston General Hospital, Launceston, TAS Australia

**Keywords:** Continuous renal replacement therapy, CRRT, Vascular access, Filter life, Femoral, Jugular, Vein

## Abstract

**Background:**

Optimising filter life and performance efficiency in continuous renal replacement therapy has been a focus of considerable recent research. Larger high quality studies have predominantly focussed on optimal anticoagulation however CRRT is complex and filter life is also affected by vascular access, circuit and management factors. We performed a systematic search of the literature to identify and quantify the effect of vascular access, circuit and patient factors that affect filter life and presented the results as a meta-analysis.

**Methods:**

A systematic review and meta-analysis was performed by searching Pubmed (MEDLINE) and Ovid EMBASE libraries from inception to 29^th^ February 2016 for all studies with a comparator or independent variable relating to CRRT circuits and reporting filter life. Included studies documented filter life in hours with a comparator other than anti-coagulation intervention. All studies comparing anticoagulation interventions were searched for regression or hazard models pertaining to other sources of variation in filter life.

**Results:**

Eight hundred nineteen abstracts were identified of which 364 were selected for full text analysis. 24 presented data on patient modifiers of circuit life, 14 on vascular access modifiers and 34 on circuit related factors. Risk of bias was high and findings are hypothesis generating.

Ranking of vascular access site by filter longevity favours: tunnelled semi-permanent catheters, femoral, internal jugular and subclavian last. There is inconsistency in the difference reported between femoral and jugular catheters.

Amongst published literature, modality of CRRT consistently favoured continuous veno-venous haemodiafiltration (CVVHD-F) with an associated 44% lower failure rate compared to CVVH. There was a trend favouring higher blood flow rates. There is insufficient data to determine advantages of haemofilter membranes.

Patient factors associated with a statistically significant worsening of filter life included mechanical ventilation, elevated SOFA or LOD score, elevations in ionized calcium, elevated platelet count, red cell transfusion, platelet factor 4 (PF-4) antibodies, and elevated fibrinogen.

Majority of studies are observational or report circuit factors in sub-analysis. Risk of bias is high and findings require targeted investigations to confirm.

**Conclusion:**

The interaction of patient, pathology, anticoagulation, vascular access, circuit and staff factors contribute to CRRT filter life. There remains an ambiguity from published data as to which site and side should be the first choice for vascular access placement and what interaction this has with patient factors and timing. Early consideration of tunnelled semi-permanent access may provide optimal filter life if longer periods of CRRT are anticipated. There remains an absence of robust evidence outside of anti-coagulation strategies despite over 20 years of therapy delivery however trends favour CVVHD-F over CVVH.

## Background

Continuous renal replacement therapy (CRRT) is a common intervention to maintain physiologic plasma composition when acute kidney injury (AKI) complicates critical illness. CRRT by definition relies on continuous blood flow through the extra-corporeal circuit to support controlled clearance of solutes and water balance. Failures of the extracorporeal circuit interrupt treatment delivery, increase cost and are potentially disruptive to other aspects of patient care.

Optimising filter life and performance efficiency in CRRT has been a focus of considerable recent research. Larger high quality studies have predominantly focussed on optimal anticoagulation strategies and this has formed the core of several reviews [1–6] with a recent meta-analysis [6, 7] favouring citrate over regional heparin to extend filter life.

Though narrative reviews exist focussing on non-anticoagulant parameters that affect filter life [1–3], no meta-analysis has ever pooled published data in this area. We performed a systematic search of the literature to identify and quantify the effect of non-anticoagulant factors and interventions that influence filter life in continuous renal replacement therapy. We have arbitrarily divided non-anticoagulant determinants of filter life into vascular access factors, circuit factors and patient factors.

Adequate vascular access allows the desired blood flow to be achieved without generating extremes of pressure between the extracorporeal circuit and the patient. Poor access results in frequent CRRT platform alarms and failure of treatment delivery or reductions in blood flow that may decrease therapy effectiveness and promote stasis with subsequent thrombosis [1–3]. Obtaining vascular access for CRRT is a frequently performed procedure. Veno-venous (VV) techniques have largely supplanted arterio-venous (AV) cannulation due to the availability and relative ease of wire-guided dual lumen catheters. However many possible combinations of vascular access catheter design, size, insertion site, inserter experience, depth of insertion and line maintenance make determining the optimal combination complex.

Patient factors such as body habitus, pathology and coagulopathy all contribute to the ease of performing CRRT and maintaining vascular access. Circuit factors include the modality of treatment with continuous veno-venous haemodialysis (CVVHD), haemofiltration (CVVH), and haemodiafiltration (CVVHD-F) all in common usage. Variation in practice also encompasses use of pre/post dilution in CVVH and CVVHD-F, target blood and fluid flow rates and circuit management practices.

## Methods

Databases of reviews were searched for similar meta-analyses and none were found. A preliminary literature search identified that the majority of studies were observational in nature being either primary observation studies or contained as sub-analyses of randomised studies. Given that our goal was to identify factors and interventions and develop hypotheses for future studies, inclusion of observational evidence was deemed acceptable. Consequently a systematic review strategy was developed following the MOOSE guideline statement for Meta-Analyses and Systematic Reviews of Observational Studies [8].

### Search strategy

The search strategy was developed by an experienced researcher (MB) and assistance provided by library staff. We searched Pubmed (MEDLINE) and Ovid EMBASE libraries to 29^th^ February 2016 with no restrictions utilising keywords, variant spellings and wildcards (Table 1). Manual review of references from included studies and potentially relevant related citations was also performed.

**Table 1:** Search Strategy

Title and Abstract Search		Title and Abstract Search/MeSH Terms
CRRT OR continuous renal replacement therapy OR CVVHD-F OR CVVD OR CVVH OR CVVHD	OR	continuous venovenous OR continuous veno-venous OR continuous veno venous
	AND	haemodiafiltration OR haemodiafiltration OR hemofiltration OR haemofiltration OR ultrafiltration
	AND	
extracorporeal circulation OR circuit* OR filter* OR vascular access OR access catheter OR catheter OR securement OR flush OR lock* OR haemofilter or haemofilter or blood flow or ultrasound OR vein	AND	safety events OR bleeding dislodgement or disconnect* OR recirculat* OR dysfunction OR failure OR life* OR interruption OR survival OR thrombosis OR clot* OR coagulant* OR “blood coagulation” [MeSH Terms]
	OR	clearance OR flux OR homeostasis OR acid base OR strong ion difference OR effectiveness OR efficacy or biocompatibility OR body habitus OR obesity OR patient position OR physiotherapy OR physical therapy OR mobilisation OR education OR training OR experience

* = wildcard search character

### Included studies

All abstracts were imported into Zotero (version 4.0.28.8, George Mason University, Fairfax, VA, USA) and duplicate entries from different databases merged. Abstracts were screened independently by two researchers (MB, EW) for potential relevance after which full text versions of the papers were obtained for all potentially relevant studies.

Studies were included if they documented filter life in hours with a comparator other than anti-coagulation intervention. All studies comparing anticoagulation interventions were searched for regression or hazard models pertaining to other variation in filter life. Abstracts detailing proceeds of meetings and conference abstracts were merged with studies by the same authors if the results were clearly duplicate entries and otherwise included. Foreign language articles were converted to English using Google Translate [9].

### Excluded studies

The following studies were excluded: Abstracts of pharmacokinetic CRRT studies, studies on cytokine mediators, micronutrients or renal recovery as well as reviews and prior meta-analyses.

We also excluded studies that solely documented filter life in a population without any comparators or relationship to independent variables, studies describing arbitrary levels of filter life without a baseline group (e.g. arbitrarily comparing survival curves between ‘short’ and ‘long’ filter life) and results that divided filter life into dichotomous time periods without specifying hours per filter or similar variations such as numbers of filters per day. In these cases authors were contacted regarding obtaining original data. Bench studies or studies involving animals were also excluded.

### Data extraction & statistical methods

Filter life and basic study data were extracted directly into Comprehensive Meta-Analysis version 3.3.070 [10] while study details were entered directly into Revman version 5.3 [11] after exporting citations from Zotero. Multiple regression covariates (using the full model where available) were converted to correlations using Rosenthal & Rubin’s r-equivalent method [12–14]; when comparison of means were also available we found this technique produced similar effect size estimates. If survival curves were presented without hazard ratios, these were extracted using methods detailed by Liu & Hanley [15–18] utilising Engauge Digitizer version 6.2 [19] and R version 3.0.2 with package survival [20, 21]. As a last resort medians and interquartile ranges were converted to means using recognised procedures and results requested from the study author [22]. One author [23] provided original data for which a survival analysis was performed in R version 3.0.2 with package survival version 2.38-3 [21] utilising a Cox proportional hazards model and further pooled with the authors (MB) previously published data [24] to produce Kaplan Meier figures.

Comprehensive Meta-Analysis version 3.3.070 [10] was utilised to present varying effect size estimates as Odds Ratios (for risk of event data) or Hazard Ratios (for time to event data) depending on the source. A random effects model was used to combine similar variables for summary effect estimates. Where a sub-group has both Hazard and Odds Ratio effect size estimates, the estimates are discussed in the text.

Quality and risk of bias was assessed utilising the GRADE approach [25] as implemented in Revman version 5.3 [11] and a summary of findings table created using GRADEpro [25]. Heterogeneity was considered on pragmatic grounds from sources of variability in the study description and statistically when sufficient comparisons were present with an I^2^ statistic <50% denoting low heterogeneity and >80% denoting high. We graded risk of detection bias for observational studies according to the trial design with a lower risk attributed to large data sets reporting multiple factors and higher risk attributed to retrospective analysis of an intervention or cohort difference.

### Classification of studies

The primary outcome was filter life. Factors associated with filter life were arbitrarily divided into patient factors, vascular access factors and circuit factors with subgroups within each level. Studies reporting multiple variables were included in more than one category.

## Results

### Search results (Fig. 1)

**Fig. 1:**
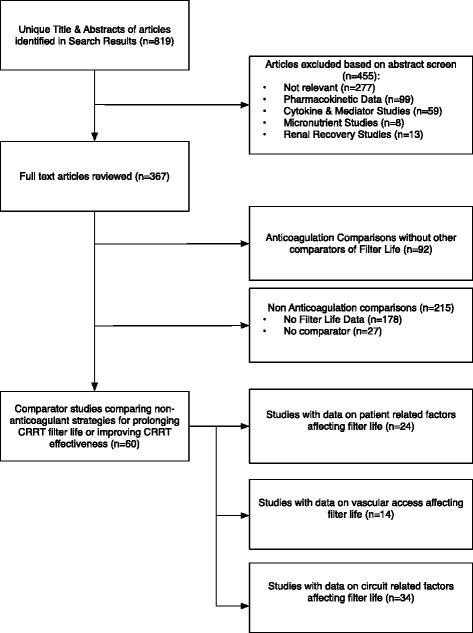
Article flow through systematic review process

**Fig. 2:**
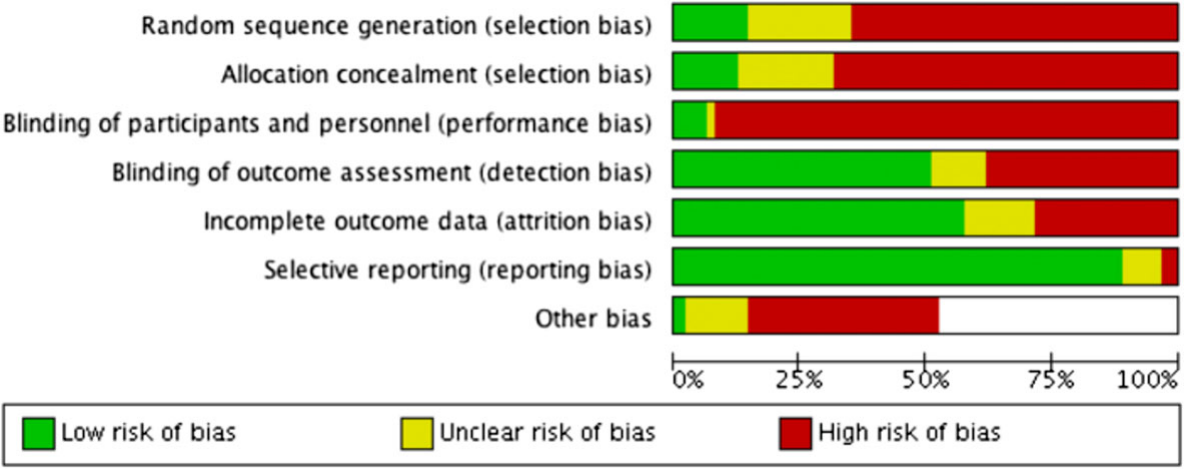
Risk of Bias Graph. For non - randomised studies detection bias risk was graded by likely influence on findings

A total of 819 abstracts were identified of which 364 were selected for full text analysis. The study selection process is outlined in Fig. 1. Characteristics of included studies are displayed in Tables 2, 3 and 4 . The most common reason for study exclusion was failure to describe filter life in hours. Figure 2 describes the overall bias summary of included studies. Bias summaries for individual studies are included with the forest plots. The authors of three studies provided clarifying information upon request and one author provided original data for reanalysis [23].

**Table 2:** Characteristics of studies reporting vascular access parameters associated with filter life [17, 19–50, 103]

Study Name	Sample Size	Design	Description	Inclusion Criteria	Exclusion Criteria	Intervention/Comparison	Control
du Cheyron et al., 2006 [35]	78 patients/193 filters	Retrospective Cohort	Antithrombin administration vs Heparin	Septic shock, CRRT, 1/2001 to 12/2004	Antithrombin cutoff parameters	Antithrombin administration	Regional Heparin
Kim et al. 2011(b) [92]	50 patients/341 circuits	Prospective Observational	Niagara vs Dolphin Catheters for CRRT	CRRT with femoral access	N/A	Niagara 13.5Fr	Controlling for other variables
Kim et al. 2011 [37]	50 patients/341 circuits	Prospective Observational	Insertion side, body position and circuit life	CRRT with femoral access	N/A	Anticoagulation	Controlling for other variables
Chua et al., 2012 [50]	71 patients/539 filters	Retrospective Cohort	Circuit Life in Hepatic Failure with AKI	Age > 18 years, Hepatic Failure, AKI, January 2006 to July 2011	N/A	Multivariate Analysis	Heparin
Morgan et al. 2012 [26]	100 patients/400 filters	Randomised Unblinded Controlled Trial	Catheter Length in CRRT	Upper body catheter, short term CRRT	exist long term catheter, indication for femoral access, systemic anticoagulation, citrate for excess bleeding risk	13.5 Fr Longer Catheter Length	Controlling for other variables
Fealy et al. 2013 [93]	46 patients/254 filters	Prospective Cohort	Comparison of Niagara & Medcomp catheters	CVVH Exposure	IHD	Medcomp 13.5Fr 24 cm O-O	Niagara 13.5Fr 24 cm D-D
Hwang et al., 2013[60]	222 patients/1236 filters	Retrospective Cohort	Nefamostat vs Heparin	CRRT treated patients 1/2004 to 12/2008	deceased within first filter use, paraquat poisoning	APTT (sec)	Controlling for other variables
Mottes et al., 2013 [38]	80 patients/343 filters	Prospective Observational	Process Control Analysis through changing model of care and education	CRRT 7/2007 to 7/2010	N/A	ICU Nurse CRRT and simulation	No Anticoagulation
Brain et al. 2014 [27]	30 patients/221 Filters	Post-hoc analysis of RCT	Citrate vs Heparin	CRRT, AKI criteria	Weight, contra-indication to study arm, pregnancy, previous CRRT, hypothermia, specific mode CRRT	Citrate Anticoagulation	Dolphin 13.5Fr
Crosswell at al. 2014 [24]	131 patients/870 filters	Retrospective Cohort	Circuit Life by Vascular Access Site	CRRT, Vascular Access Data	N/A	Vascular Access Site Comparison	13.5 Fr Shorter Catheter Length
Dunn & Sriram, 2014 [23]	355 patients/1332 filters	Retrospective Cohort	Patient and Treatment Variables on Filter Life	CRRT 1/2008 to 8/2011	N/A	Multivariate Analysis	Nephrology nurse run CRRT
Sponholz et al., 2014 [94]	1621 patients/10643 filters	Retrospective Cohort	Anticoagulation strategies over 5 year period	CRRT, 1/2007 to 12/2012	N/A	Femoral Access	Non-Femoral Access
Wang et al. 2014 [36]	34 patients/126 filters	Prospective Cohort Study	Effect of mobilisation on filter life	CRRT, 8/2011 to 8/2012, temporary vascular access	Permanent vascular access, SLED	Mobilisation	Baseline
Zhang et al., 2015 [39]	23 patients/79 filters	Prospective Observational	Patterns and Mechanisms of artificial kidney failure	Age > 18 years, CRRT, Prismaflex	Non-prismaflex machine, plasma exchange, CRRT duration <24 hours	Stratify filter failure by access pressure	Controlling for other variables

**Table 3:** Characteristics of studies reporting circuit life parameters associated with filter life

Study Name	Patients/Filters	Design	Description	Inclusion Criteria	Exclusion Criteria	Intervention/Comparison	Control
Martin et al., 1994 [95]	255 patients/1306 filters	Retrospective Cohort	Stratified anticoagulation and filter function	CRRT		Polyacrylonitrile (AN69) Membrane	Polyamide (FH66) Membrane
Baldwin et al. 1996* [40]	12 patients/38 filters	Randomised Cross Over	Membrane Type Comparison	N/A	N/A	Hollow Fiber Membrane	Flat Plate Membrane
Baldwin et al. 1996 (2)* [55]	14 patients/54 filters	Randomised Cross Over	Regional anti-coagulation with one or two heparin sites	N/A	N/A	Single Site Heparin Delivery	Double Site Heparin Delivery
Leslie et al., 1996 [56]	18 patients/105	Randomised Cross Over	Compare circuit heparin delivery site in CVVHD	CRRT	thrombocytopenia, anticoagulation indication, haemorrhage, coagulopathy	Access Line Heparin Delivery	Pre-Filter Heparin Delivery
Reeves et al. 1997 [58]	40 patients/91 filters	RCT - Unblinded	Circuit priming with heparin or albumin plus heparin on filter life	AKI requiring CRRT	Coagulopathy, DIC, HITS, albumin hypersensitivity	Albumin Prime	Saline Prime
Ramesh Prasad et al., 2000 [53]	34 patients/130 filters	RCT - Unblinded	Comparison of high blood flow with frequent saline flushes vs low blood flow and hourly flushes on filter life			Qb 200-250 ml/min & 30 min flush	Qb 125 ml/min & 60 min flush
Uchino et al., 2003 [96]	48 patients/309 filters	Prospective Observational	Pre vs Post Dilution CVVH on Filter Life	2/2001 to 7/2002. All CRRT surviving >24 hours		Pre-dilution CVVH	Post Dilution CVVH
Rickard et al. 2004 [59]	51 patients/60 filters	RCT - Unblinded	CRRT Fluid warmer or not on patient hypothermia	>18 yrs., baseline temperature 36–39.5 degrees C	malignant hyperthermia, other external warming/cooling, burns	Fluid Warmer	No Fluid Warmer
Egi et al., 2005 [97]	63 patients/246 filters	Prospective Observational	Filter patency differing blood flow, CVVH and citrate dose	CRRT requirement, short filter life with heparin, high risk bleeding	Hepatic failure/hepatitis	CVVH Qb:150 ml/min Citrate 14 mmol/L, iCa 1.0-1.2	CVVH Qb:200 ml/min Citrate 11 mmol/L, iCa 1.1-1.3
van der Voort et al., 2005 [49]	16 patients/32 Filters	Randomised Cross-Over	Pre vs Post Dilution CVVH on Filter Life	Mechanical ventilation, AKI, no prior CRRT	specific CVVH mode, active bleeding, planned surgery	CVVH Predilution	CVVH Post-Dilution
de Pont et al. 2006 [48]	8 patients/15 filters	Randomised Cross Over	Pre-dilution vs Post-dilution	CRRT indication	recent bleeding, time limits on last exposure to aspirin, UFH, LMWH, coagulopathy	CVVH Pre-dilution & Qb 140 ml/min	CVVH Post-Dilution & Qb 200 ml/min
Pichaiwong et al. 2006 [42]	17 patients/17 filters	Prospective Observational	Comparison of two haemofilters performance and biocompatibility	AKI, CVVH		Cellulose Sureflex150E	Synthetic Polysulfone AV-400
Ricci et al., 2006 [47]	15 patients / 30 filters	Prospective Observational	Convective vs Diffusive Solute Clearance	AKI, CRRT		CVVH	CVVHD
Davies et al. 2008 [46]	31 patients/31 circuits	Randomised Cross Over	CRRT Mode Comparison	>18, CRRT	Contraindication to heparin, thrombocytopenia	CVVH	CVVHDF
Kim et al. 2010 [63]	30 patients/140 filters	Retrospective Cohort	Circuit clotting due to mechanical failure	CRRT with femoral access		Mechanical Failure	Non-Mechanical Failure
Nagarik et al., 2010 [61]	65 patients/65 filters	Prospective Observational	Comparing heparin with saline circuit flushes	AKI requiring CRRT		Saline Circuit Flushes	Heparin Infusion
Kim et al. 2011 [37]	50 patients/341 circuits	Prospective Observational	Insertion side, body position and circuit life	CRRT with femoral access		CRRT Mode (CVVH)	CVVHDF
Nurmohamed et al., 2011 [98]	63 patients/243 filters	Retrospective Cohort	Predilution vs Postdilution CRRT	AKI requiring CRRT, 11/2004 to 12/2006	Single filter < 12 hours duration	CVVH Predilution	CVVH Post-dilution
Panphanphoet et al., 2011 [62]	121 patients/121 filters	Retrospective Cohort	Saline flush to prevent filter failure	AKI requiring CRRT 1/2004 to 12/2006		Saline Flushing	No Saline Flushing
Baldwin et al. 2012 [57]	38 patients/80 filters	Prospective Pre-Post Cohort	Horizontal vs Vertical Bubble Trap orientation	N/A	N/A	Horizontal Bubble Trap	Vertical Bubble Trap
Chua et al., 2012 [50]	71 patients/539 filters	Retrospective Cohort	Circuit Life in Hepatic Failure with AKI	Age > 18 years, Hepatic Failure, AKI, January 2006 to July 2011	N/A	Multivariate Analysis	Controlling for other variables
Eastwood et al., 2012 [54]	21 patients/41 filters	Prospective Observational	Haemodynamic impact of slower pump speed	Convenience sample, age > 18, CRRT requirement, weekdays only	N/A	Slow initial pump speed	Normal initial pump speed
Schetz et al. 2012 [43]	39 patients/151 filters	RCT - Blinded	AN69 ST (surface treated) Haemofilter vs non-ST Haemofilter	CRRT, 30 kg - 120 kg	HIT, pregnancy, indication for systemic anticoagulation, poor short term prognosis	AN69ST Membrane (Surface Treated)	AN69 Membrane
Hwang et al., 2013 [60]	222 patients/1236 filters	Retrospective Cohort	Nefamostat vs Heparin	CRRT treated patients 1/2004 to 12/2008	deceased within first filter use, paraquat poisoning	APTT (sec)	Controlling for other variables
Morabito et al., 2013 [99]	40 patients/240	Prospective Observational	RCA-CVVHDF vs baseline RCA-CVVH	CRRT > 72 hrs, AKI post cardiac surgery, 5/2012 to 12/2012	Contra-indication to citrate	CVVH Prismocitrate 10/2 + Prismasol	CVVHDF Prismocitrate 18 + Phoxilium
Mottes et al., 2013 [38]	80 patients/343 filters	Prospective Observational	Process Control Analysis through changing model of care and education	CRRT 7/2007 to 7/2010	N/A	ICU Nurse CRRT and simulation	Nephrology nurse run CRRT
Bonassin et al., 2014 [45]	53 patients/66 filters	Retrospective Cohort	Membrane Area/Size Comparison	Consecutive CRRT 11/2007 to 6/2009	N/A	Filter AV 1000S	Filter AV 600S
Dunn & Sriram, 2014 [23]	355 patients/1332	Prospective Observational	Patient and Treatment Variables on Filter Life	CRRT 1/2008 to 8/2011	N/A	Multivariate Analysis	Controlling for other variables
Fu et al., 2014 [41]	425 patients/unclear	Prospective Observational	Prognostic model of circuit life	1/2011 to 2/2013, CRRT, age > 18, CRRT > 24 hrs, available blood analysis	<72 hr post cardiac surgery, ECMO Rx	Multiple Regression	
Page et al., 2014 [100]	152 patients/401 filters	Retrospective Cohort	System change with citrate, education	CRRT usage 1/2009 to 12/2012		Citrate + Education + Survival Plan + Lower Dose + CVVHD	Heparin + CVVH
Wang et al. 2014 [36]	34 patients/126 filters	Prospective Observational	Effect of mobilisation on filter life	CRRT, 8/2011 to 8/2012, temporary vascular access	permanent vascular access, SLED	Mobilisation	Baseline
Choi et al., 2015 [30]	60 patients/101 filters	RCT - Unblinded	Nafamostat vs No Anticoagulation	CRRT, High bleeding risk	Pregnancy, Allergy to nafamostat, Hypercoagulable states	Nafamostat	No Anticoagulation
Yin et al. 2015 [44]	17 patients/68 filters	RCT - Blinded	Comparison of two haemofilter membranes	Age > 16 yrs., weight 30-120 kg		AN69 ST100	AN69 M100
Ede & Dale, 2016 [101]	78 patients/118 filters	Retrospective Pre-Post Cohort	CRRT Effectiveness and Circuit Life between CVVH and CVVHDF	All CRRT pre/post 9/2012, age > 18,	N/A	CVVHDF	CVVH

**Table 4:** Characteristics of included studies reporting patient factors by date of investigation

Study Name	Patients/Filters	Design	Description	Inclusion Criteria	Exclusion Criteria	Intervention/Comparison	Control
Stefanidis et al., 1995 [73]	60 patients/270 filters	Retrospective Observational	Multivariate analysis of hematologic and hemostatic variables on filter life	N/A	N/A	Multivariate Analysis of filter life by underlying hemostatic factor	Controlling for other variables
de Pont et al., 2000 [31]	32 patients/12 filters	Randomised Double Blind Cross-over	Nadroparin vs Dalteparin Anticoagulation	CRRT indication	time limits on recent UFH, LMWH, bleeding, coagulopathy	Nadroparin	Dalteparin
Ramesh Prasad et al., 2000 [53]	34 patients/130 filters	RCT - Unblinded	Comparison of high blood flow with frequent saline flushes vs low blood flow and hourly flushes on filter life	All patients receiving CRRT	N/A	Qb 200-250 ml/min & 30 min flush	Qb 125 ml/min & 60 min flush
Uchino et al., 2003 [96]	48 patients/309 filters	Prospective Observational	Pre vs Post Dilution CVVH on Filter Life	2/2001 to 7/2002. All CRRT surviving >24 hours	N/A	Pre-dilution CVVH	Post Dilution CVVH
Kutsogiannis et al., 2005 [33]	31 patients/79 filters	Randomised Controlled Trial	Citrate vs Heparin for CRRT	age > 18 yr, AKI, CRRT	contra-indication to heparin/citrate, indication for systemic heparin	Antithrombin III activity, IU/mL	Summary - AT levels
Bouman et al., 2006 [72]	10 patients/10 filters	Prospective Observational	Pre-post blood sampling of clotting mediators	CRRT in ICU	coumarins,platelet inhibitors, UFH/LMWH, CRRT within timelimits of study; discontinuation of CVVH othan than clotting	Prothrombin Fragment F1 + 2 Elevation	No F1 + 2 Elevation
du Cheyron et al., 2006 [35]	78 patients/193 filters	Retrospective Cohort	Antithrombin administration vs Heparin	Septic shock, CRRT, 1/2001 to 12/2004	Antithrombin cutoff parameters	Antithrombin administration	Heparin
Lasocki et al., 2008 [74]	28 patients/28 filters	Retrospective review	Anti-PF4/heparin antibodies & CRRT filter clotting	11/2004 to 5/2006, frequent filter clotting, anti-PF4/heparin antibody presence	0	Danaparoid	Heparin/PF4 Antibodies
Ghitescuet et al., 2009 [65]	77 patients/77 filters	Retrospective Cohort	Correlation between sepsis and filter failure	CVVH patients, 7/2001 to 9/2005	thrombocytopenia, bleeding < 24 hrs	Sepsis/Severe Sepsis	No Sepsis
Oudemans-van Straaten et al., 2009 [34]	14 patients/unclear	Randomised Cross-over Design	CVVH at 2 L/4 L flow effect on ant--Xa levels and coagulation	Adult, AKI, CRRT	High bleeding risk, HITS, indication for therapeutic anticoagulation	CVVH at 4 L flow	CVVH at 2 L flow
Zick et al., 2009 [68]	24 patients/98 filters	Prospective, observational study, non-randomised.	Citrate anticoagulation in liver failure: comparison of two groups stratifed by bilirubin	CRRT with anticoagulation to heparin or high risk of bleeding	N/A	bilirubin > 3 mg/dL	bilirubin < 3 mg/dL
Kim et al. 2010 [63]	30 patients/140 filters	Retrospective Observational	Circuit clotting due to mechanical failure	CRRT with femoral access	N/A	Mechanical Failure	Non-Mechanical Failure
Kiser et al., 2010 [32]	10 patients/40 filters	Prospective, randomized, double blind	Efficacy and Safety of bivalirudin vs heparin in CVVH	age > 18 yr, AKI, CRRT without anticoagulation, filter life <24 hr	contra-indication to heparin, bivalirudin, ESRF, IHD, pregnancy, aPC, prostacyclin, indication for therapeutic anticoagulation, active hemorrhage risk	Antithrombin III activity	Controlling for other variables
Kim et al. 2011[37]	50 patients / 341 circuits	Prospective, non-randomised, observational.	Insertion side, body position and circuit life	CRRT with femoral access	N/A	Anticoagulation	No Anticoagulation
Kim et al. 2011 (b) [92]	50 patients/341 circuits	Prospective, non-randomised, observational.	Niagara vs Dolphin Catheters for CRRT	CRRT with femoral access	N/A	Niagara 13.5Fr	Dolphin 13.5Fr
Chua et al., 2012 [50]	71 patients/539 filters	Retrospective Cohort	Circuit Life in Hepatic Failure with AKI	Age >18, Hepatic Failure, AKI, January 2006 to July 2011	N/A	Multivariate Analysis	Controlling for other variables
Saner et al., 2012 [102]	68 patients/68 filters	Observational	Citrate in liver transplant recipients	Consecutive Liver transplant recipients 11/2004 to 9/2007, AKI	N/A	Septic	Non Septic
Zhang et al., 2012 [64]	54 patients/255 circuits	Prospective observational study, non-randomised.	Variables associated with circuit life span	Age > 18y, CVVH, ICU LOS > 72 hours	Pregnant, age >80, contra-indication to heparin, HITT, high bleeding risk	Multivariate Analysis	Controlling for other variables
Brunner et al. 2013 [67]	16 patients/37 filters	Prospective physican choice AT3/Heparin. Retrospective Analysis	CRRT in Hepatic Failure with AKI	Physician Choice	N/A	Antithrombin administration	Heparin
Fealy et al. 2013 [93]	46 patients/254 filters	Prospective Cohort	Comparison of Niagara & Medcomp catheters	CVVH	IHD	Medcomp 13.5Fr 24 cm O-O	Niagara 13.5Fr 24 cm D-D
Hwang et al., 2013 [60]	222 patients/1236 filters	Retrospective Cohort	Nefamostat vs Heparin	CRRT treated patients 1/2004 to 12/2008	deceased within first filter use, paraquat poisoning	APTT (sec)	Controlling for other variables
Dunn & Sriram, 2014 [23]	355 patients/1332	Retrospective Cohort	Patient and Treatment Variables on Filter Life	All CRRT 1/2008 to 8/2011	N/A	Multivariate Analysis	Controlling for other variables
Fu et al., 2014 [41]	425 patients/425 filters	Prospective cohort	Prognostic model of circuit life	1/2011 to 2/2013, CRRT, age > 18, CRRT > 24 hrs, available blood analysis	<72 hr post cardiac surgery, ECMO Rx	Multivariate Analysis	Controlling for other variables
Wang et al. 2014 [36]	34 patients/126 filters	Prospective Cohort Study	Effect of mobilisation on filter life	8/2011 to 8/2012, CRRT, temporary vascular access	permanent vascular access, SLED	Filter Life in Mobilisation	Baseline Filter Life
Choi et al., 2015 [30]	60 patients/101 filters	Unblinded RCT	Nafamostat vs No Anticoagulation	CRRT, High bleeding risk	Pregnancy, Allergy to nafamostat, Hypercoagulable states	Nafamostat	No Anticoagulation

Fourteen studies contain filter life data related to vascular access properties. None of these were of high quality. Only one had a randomised design where the primary outcome was filter life [26], while a second randomised trial presented a post-hoc analysis of filter life by vascular access site [27]; neither was blinded. Two high quality randomised trials were identified in the literature search that did not report filter life related to vascular access but to catheter function; these are discussed in the relevant sections [28, 29].

Thirty-four studies contain filter life data related to circuit factors of which 13 had a prospective randomised design but only two were blinded. Twenty-four studies contain filter life data related to patient variables however the majority of indices of filter life were from sub-analyses. No large high quality randomised studies existed. Only five studies in this analysis had a prospective randomised design [30–34] and of these only one described blinding of investigators.

### Overall filter life

Amongst included studies from the entire systematic review where mean filter life was available, overall mean filter life was 21.92 h (*n* = 7502, SD = 10.89).

### Vascular access factors and filter life

#### Access site and filter life

**Fig. 3:**
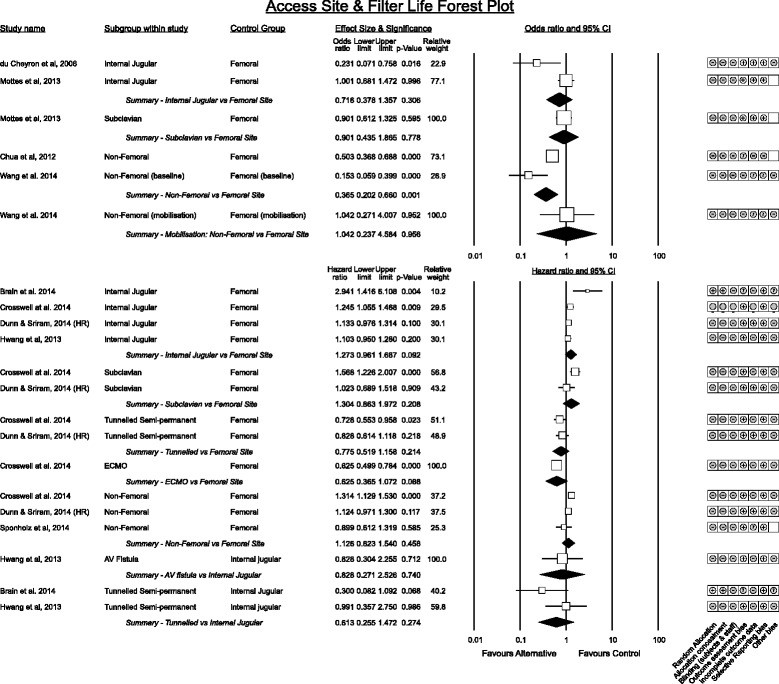
Vascular access insertion site association with filter life. Effect estimates are grouped by category. Studies reporting as difference in means, correlations or risk are summarised in odds ratios. Hazard ratios are presented separately

Figure 3 displays grouped effect estimates for comparisons between vascular access sites. The femoral vein was the most common vascular access site utilised across studies that contained filter life data. Significant dispersion of estimates exists between studies comparing femoral and non-femoral access routes. No data existed on the order of catheter insertion sites in individual patients.

Of studies reporting internal jugular vs. femoral catheter sites, du Cheyron [35] in 78 patients favoured internal jugular access whereas the grouped effect estimate of studies reporting time to event outcomes trended toward the femoral site with an associated 27% (95% CI −4% to 69%, *p* = 0.092) increase in filter survival with low heterogeneity (I^2^ = 24.8%).

Results comparing subclavian vs femoral catheters also suggested a trend associating improved filter life from femoral access whereas tunnelled semi-permanent catheters and direct connection to ECMO circuits were associated with longer filter life when compared to femoral access. Again statistical significance was influenced by choice of statistical model for the pooled effect with fixed effects models reaching significance.

**Fig. 4:**
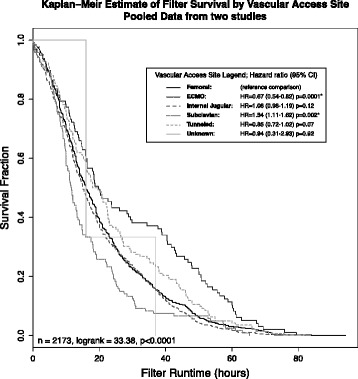
Pooled survival analysis of filter life by vascular access site from 2173 filters from two studies [23, 24]. * signifies curves that demonstrate a statistically significant difference to femoral access. Sites denote temporay catheters except for tunnelled semi - permanent devices and ECMO

Datasets made available from two studies [23, 24] were pooled to construct survival curves by filter site for 2173 filters. Subclavian access was associated with significantly worse filter life than femoral access whereas temporary internal jugular catheters was no different. Tunnelled access (14.5Fr) trended toward longer filter life while a direct connection to ECMO provided the longest filter life (Fig. 4).

Though not reporting filter life, an important sub-analysis of a large multi-centre study of CRRT dose [29] suggested femoral catheters had limited impact on CRRT dose delivery compared to non-femoral catheters.

A single small study described patient activity and catheter site with filter life; Wang et al. [36] found at baseline non-femoral catheters were associated with longer filter life however this advantage did not persist with mobilisation.

#### Access side and filter life (Fig. 5)

**Fig. 5:**
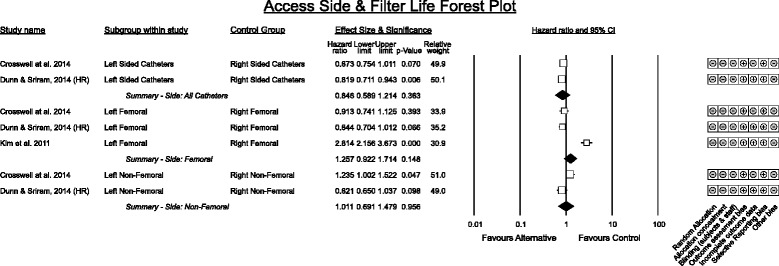
Access side and Filter Life. HR denotes original study data was re - analysed for meta - analysis with a Cox proportional hazards model

Three studies provided comparison data on the side of vascular access. Results for femoral side were heterogeneous (I^2^ = 82.8%) with the study by Kim [37] a significant outlier.

Of relevance to side selection is a multicentre randomised control trial that investigated time to catheter dysfunction as an outcome rather than filter life [28] between right or left jugular and femoral placement in intermittent haemodialysis and/or CRRT. This study found no significant difference in risk of dysfunction between right jugular and femoral sites (trend favoured right jugular) however left jugular performed significantly worse (adjusted hazard ratio vs femoral 1.89 (95% CI 1.12 – 3.21, *p* < 0.02)).

#### Catheter properties associated with filter life (Fig. 6)

**Fig. 6:**
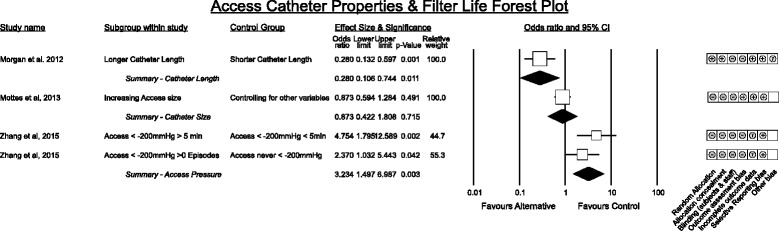
Catheter Properties associated with filter life

A single randomised comparison of catheter length [38] in great thoracic veins with confirmation of atrial tip placement vs superior vena cava tip positioning demonstrated superiority of longer (atrial) catheters. Importantly this study reported no difference in incidence of atrial or ventricular arrhythmias between the two catheter lengths.

Only one study in a paediatric population reported catheter size comparisons in relation to filter life [26] demonstrating only a weak signal. However the effect is likely greater given the report of a sub-analysis of the large RENAL dataset [29] demonstrating achievement of increased renal dose with larger catheters.

Extremely negative access pressures were associated with decreased filter life [39] in a single study.

#### Catheter types association with filter life

**Fig. 7:**
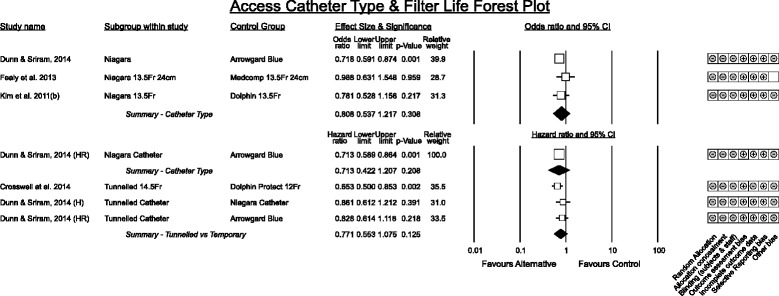
Catheter type. Arrowgard Blue denotes a group comprising 16 cm or 20 cm 12Fr or 25 cm 14Fr polyurethane antimicrobial treated catheters. Niagara when not specified denotes a group comprising 15 cm, 20 cm or 24 cm 13.5Fr polyurethane catheters. HR denotes reanalysis of the original data by Cox proportional hazards model

All studies reporting differences between temporary vascular access devices used a before-after design in an intensive care unit. Across all studies a trend favouring the Niagara catheter (Bard Canada) being associated with longer filter life was observed however the pooled effect was not significant. Tunnelled cuffed semi-permanent devices trended towards superior filter life compared to temporary devices in each analysis (Figs. 3, 4, and 7) – often these catheters have larger diameters (14.5Fr to 15.5Fr).

### Circuit factors associated with filter life

#### Haemofilter membrane characteristics (Fig. 8)

**Fig. 8:**
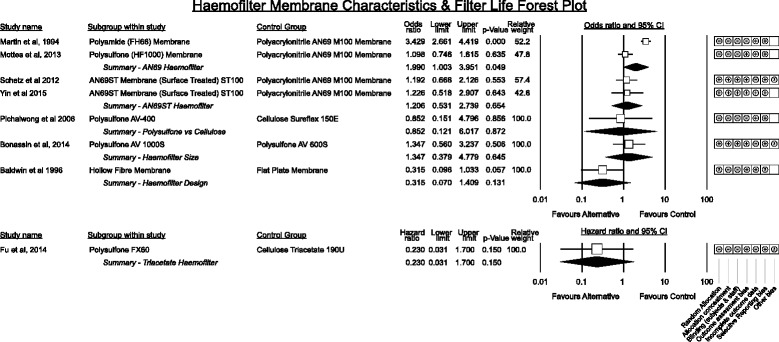
Grouped effect estimates of haemofilter membrane characteristics associated with filter life

Studies comparing haemofilter membranes spanned several advancements in membrane technology over a 22-year period. One early randomised study [40] demonstrated an advantage of hollow fibre membranes over flat plate haemofilter configurations for filter life while the remainder analysed contemporary hollow fibre membranes. Numbers of studies for each membrane comparison were small.

A trend favouring polysulfone membranes ahead of cellulose triacetate in being associated with longer filter life was apparent in one multiple regression analysis [41] but a newer modified cellulose membrane showed no difference [42]. No significant difference in filter life existed between the non-surface coated AN69 membrane and a polysulfone membrane in one study [38]. Interestingly newer surface treated (heparin binding and potentially more biocompatible) AN69ST membrane did not show any advantage in filter life over the non-surface treated AN69 in two randomised studies [43, 44].

Classically diffusive transport improves with haemofilter membrane area and anecdotally increased membrane area prolongs filter life however a single study comparing membrane area did not demonstrate an advantage [45].

#### CRRT mode, Pre vs post dilution and CRRT dose (Fig. 9)

**Fig. 9:**
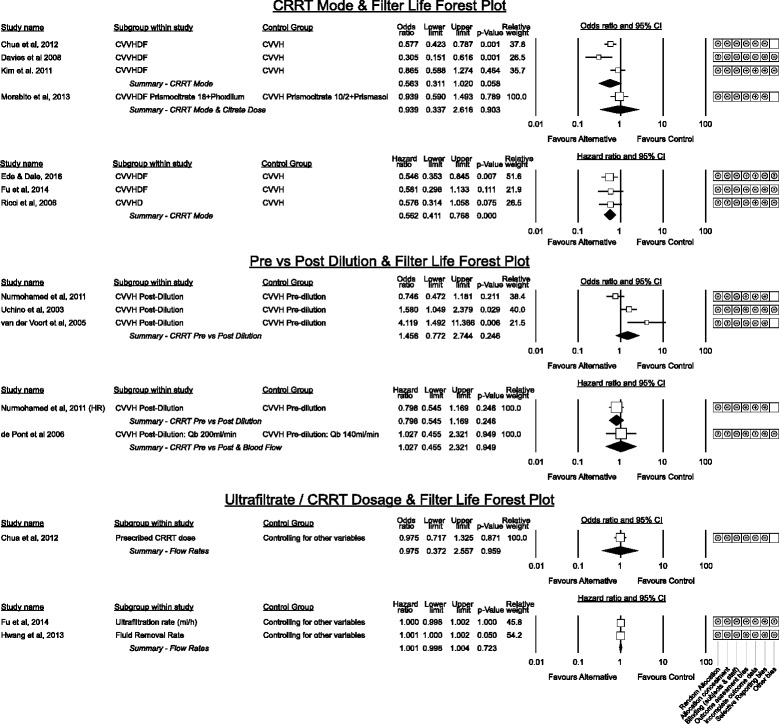
Grouped estimated effect sizes for CRRT Modes, Pre vs Post Dilution CVVH and CRRT Fluid Dosing in relation to filter life

The optimum modality of CRRT for filter life was consistent across nearly all studies including two randomised [46, 47] trials with CVVHD-F (or CVVHD [47]) associated with a 44% lower failure rate compared to CVVH (Fig. 9). Statistical heterogeneity was low (I^2^ for event risk comparisons 25.5%, *p* = 0.058; hazard ratio comparisons zero) and the result reached significance in time to event analysis (*p* < 0.001) with minimal dispersion.

Whether CVVH with pre or post dilution is superior was less clear with more heterogeneity (I^2^ = 48.6%) across risk results and no overall trend (*p* = 0.245). Of the two small randomised studies in this group [48, 49] only van der Voort et al. purely compared pre vs post dilution and favoured pre-dilution to prolong filter life.

Higher CRRT dosage, prescribed ultra-filtration rate and fluid removal rate were not associated with differences in filter life amongst the retrospective analyses that reported this outcome [36, 41, 50]. Although not directly reporting filter life, the results of two large multi-centre trials of RRT intensity are relevant in regards to CRRT dose suggesting that higher intensity RRT may be associated with decreased filter life; the RENAL Investigators [51] used 0.93 ± 0.86 filters per day in the high intensity group vs 0.84 ± 0.81 in the lower group (*p* < 0.001). Similarly Palevsky et al. [52] report 3178 CRRT treatments in 563 patients in the intensive arm vs 2789 in 561 patients in the lower intensity group.

#### Blood flow and filter life (Fig. 10)

**Fig. 10:**
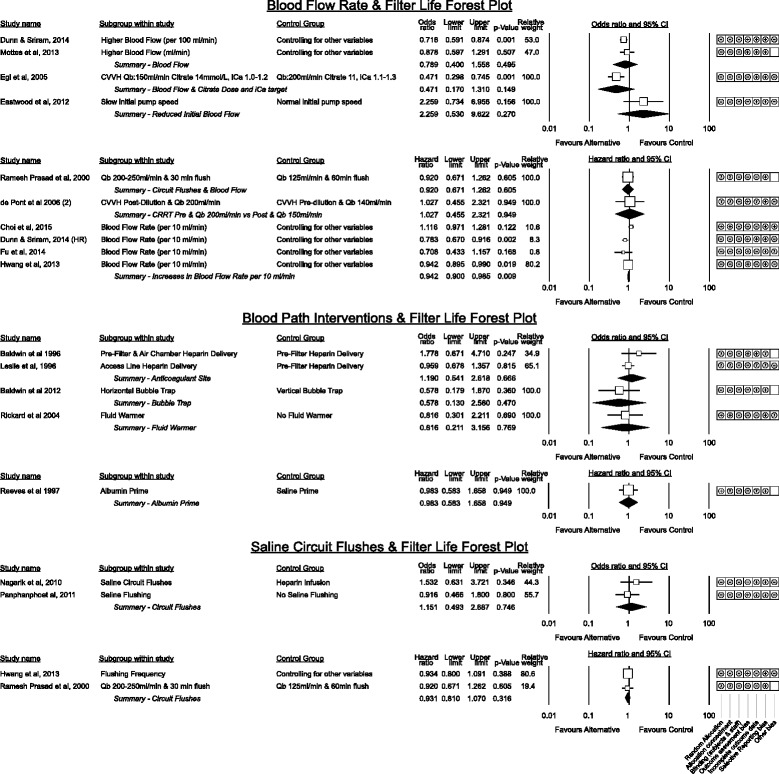
Grouped estimated effect sizes of blood flow and blood pathway interventions associated with filter life. HR denotes the re - analysis of Dunn and Sriram, 2014 [23] using a Cox proportional hazards model. Qb denotes blood flow

Higher blood flow rates have been hypothesised to prolong filter life by minimising stasis within the blood path [3] however results vary across studies. Of the three studies with randomised design none detected a difference though blood flow was not the primary outcome in the comparison by Choi et al. [30] and the studies by Ramesh Prasad et al. [53] and de Pont et al. [48] did not solely assess blood flow.

Though Mottes [38] did not show a significant association between blood flow and filter life, the pooled effect of studies contributing to the hazards model favoured higher blood flow (HR = 0.942, *p* = 0.009, I^2^ = 25.8%). This equates to a 5.8% increase in filter survival for each 10 ml/min increase in blood flow however as none of these studies were designed to directly compare low and high blood flow, this result should be considered supportive only.

The single study [54] investigating a gradual step up of initial blood pump speed to 200 ml/min over a shorter 4 min period vs a 10 min period found no benefit on patient haemodynamic parameters and a trend toward worse filter life at the slower step up.

#### Blood path interventions associated with Filter Life

Two small studies showed no benefit from different sites of heparin delivery [55, 56]. No benefit was observed using a modified horizontal bubble trap on the return line [57], priming the circuit with albumin before use [58] to improve biocompatibility or by the presence or absence of a fluid warmer on the return blood path [59].

Saline flushing of the circuit was not beneficial however the only randomised study [53] simultaneously compared differences of blood flow. The remaining studies [60–63] utilised saline flushes in patients with a coagulopathy that contra-indicated use of heparin.

#### CRRT system management and staff education interventions (Fig. 11)

**Fig. 11:**
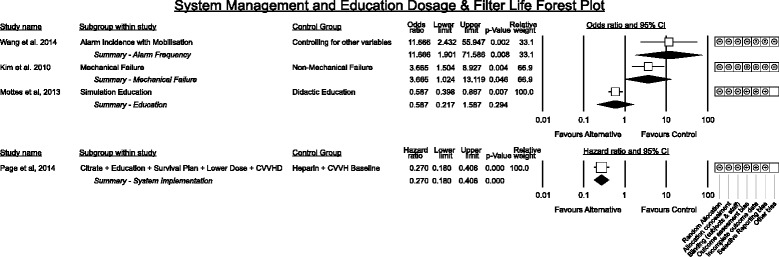
Grouped effect estimates of system management factors and education interventions associated with filter life

The number of alarms and an arbitrary definition of CRRT mechanical failure were both associated with worse filter life in two small studies [36, 63]. Education around overall CRRT management with a structured simulation event was beneficial in two studies though only the study by Mottes et al. [38] purely assessed focussed education alone without other interventions.

### Patient factors associated with filter life

#### Patient factors

**Fig. 12:**
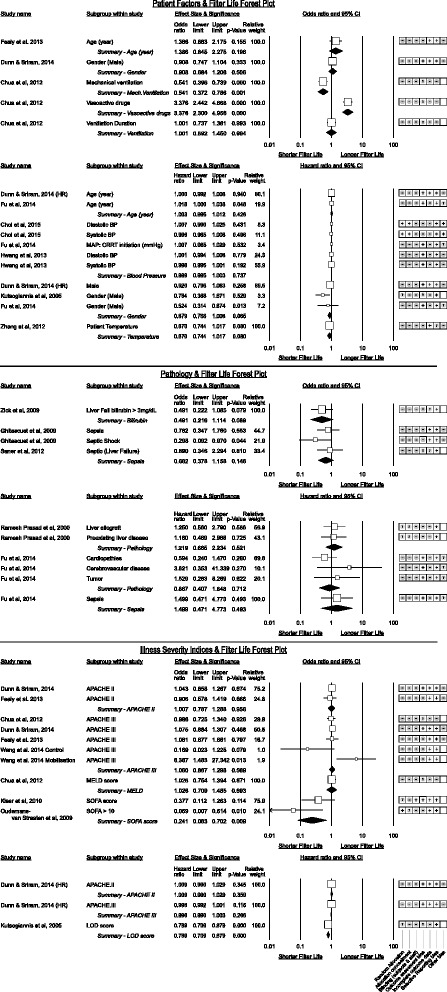
Baseline patient data, pathology and illness severity associations with CRRT filter life. Effect estimates are grouped by category. Studies reporting as difference in means, correlations or risk are summarised in odds ratios. Hazard ratios are presented separately

Figure 12 presents grouped effect size estimates for patient factors associated with CRRT filter life. Among baseline factors increasing patient age and blood pressure were not associated with a difference in filter life however male sex trended toward shorter filter life with the pooled hazard estimate nearly reaching statistical significance though heterogeneity was moderate (*p* = 0.065, I^2^ = 54.4%).

Increases in body temperature demonstrated a non-significant signal toward association with shorter filter life in one study with a 14.9% (95% C.I. −1.7% to 34.4%) increase in the failure rate per degree Celsius elevation [64]. Another single study suggested that presence of mechanical ventilation was associated with worse filter life while vaso-active drug therapy was an advantage [50].

#### Patient pathology (Fig. 12)

The most common pathology reported was sepsis. In general studies reported sepsis to have a negative effect on filter life with Ghitescuet [65] suggesting septic shock and sepsis fall on a continuum to reduce filter life further however the effect did not reach significance.

Filter life in the setting of liver failure with bilirubin > 3 mg/dL nearly reached significance for being associated with worse filter life. Not included in the forest plot due to the number of contrasts and no attempt to control for other variables is a study by Agarwal et al. [66] which contrasted anticoagulation free filter life in coagulopathic patients with acute liver failure, decompensated chronic liver disease, post liver transplant recipients, sepsis or haematological disorders. They found that haematological disorders had significantly longer filter life (x̄=21.7 ± 19.7 h) however all other groups demonstrated poor filter life with mean duration less than 12 h. The most comprehensive description of patient factors interacting with filter life in acute liver failure or decompensated liver disease is found in Chua et al. [50] where MELD score, APTT, bilirubin, mechanical ventilation, platelet count and INR were associated with filter life in this population. Discussions regarding filter life and anti-coagulation in liver failure patients requiring CRRT are the subject of several studies [50, 66–71] and growing support is emerging for the safety and efficacy of citrate in this population [70].

#### Illness severity (Fig. 12)

Illness severity scoring systems demonstrated heterogeneous association with filter life effect. The summary effect for increasing APACHE II and III (I^2^ = 57.2%) scores demonstrated no association with filter life however higher SOFA scores and higher LOD scores were associated with decreased filter life in isolated studies.

#### Biochemical parameters (Fig. 13)

**Fig. 13:**
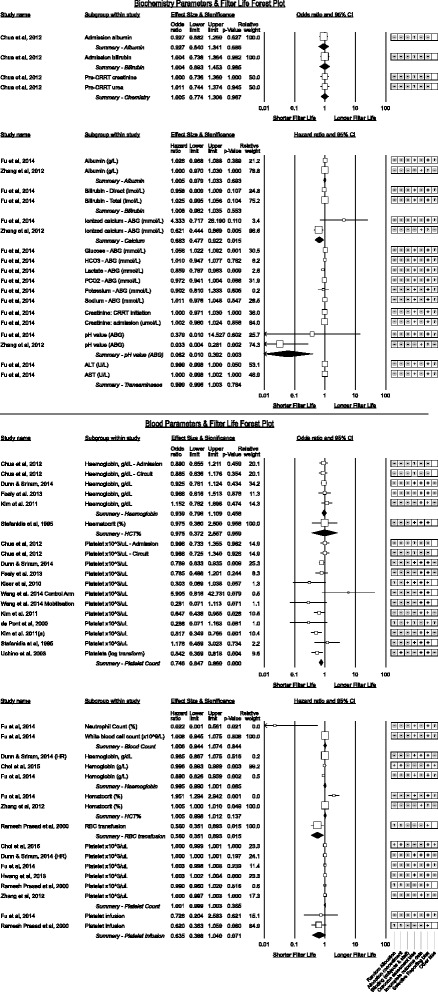
Biochemistry and blood parameters associated with CRRT filter life

Only two studies displayed sub-analyses of biochemical abnormalities in association with filter life [41, 64]. A single study suggested lactate concentration displayed an association between high lactate and shorter filter life with a 14% (95% C.I. 3%–23%) increase in failure rate per 1 mmol/L increase in lactate.

Two studies suggested higher ionised calcium was associated with shorter filter life (neither study was using citrate anticoagulation) however the effect direction was not consistent.

Lower pH was associated with longer filter life equating to a 59% (95% C.I. 10% to 59%) increase in filter survival rate per 0.1unit fall in pH. Zhang et al. [64] also demonstrated statistical significance between Kaplan-Meier curves for pH above or below 7.35 however interactions between pH and other patient factors are not explored.

#### Blood parameters (Fig. 13)

Blood count parameters demonstrated no significant association between haemoglobin (or haematocrit) measurements and filter life. There was inconsistency between higher platelet counts and shorter filter life; amongst results reported as odds ratios the summary effect for higher platelet counts reached statistical significance though heterogeneity was moderate (*p* < 0.0005, I^2^ = 53.5%) whereas the result for studies reporting a hazard ratio was not significant. A single study suggested higher neutrophil counts were associated with decreased filter life [41].

Receipt of a platelet or packed red cell infusion were both associated with a reduction in filter life though platelet infusion did not quite reach statistical significance.

#### Coagulation parameters (Fig. 14)

**Fig. 14:**
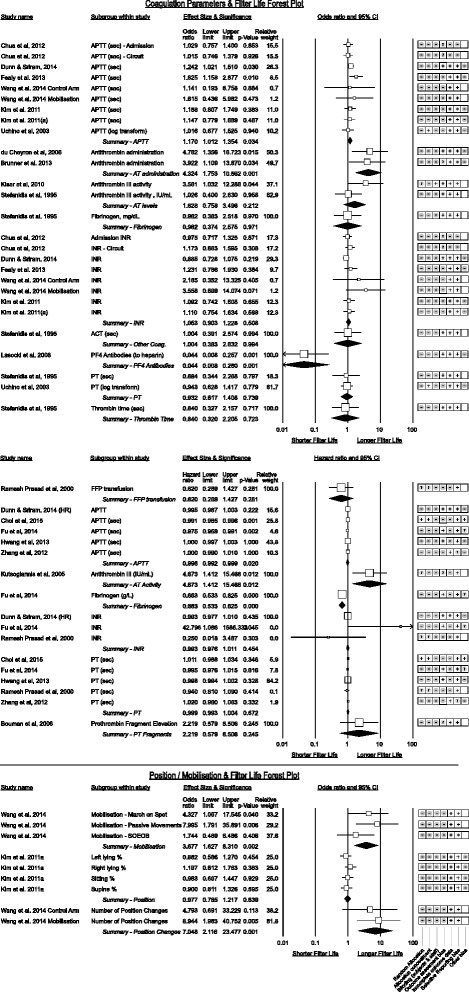
Coagulation and Position/Mobilisation parameters associated with CRRT filter life

A positive association between APTT and filter life was observable amongst studies reporting as odds ratio studies (*p* = 0.034) with a 1 s increment increasing the odds of filter survival by 1.17. However an inconsistent trend toward shorter filter life reached significance for pooled hazard ratios though clinically insubstantial (odds of failure increase by 1.004 for each second increase). Heterogeneity for APTT was moderately high I^2^ = 75.9%. INR and prothrombin time (PT) subgroups were inconsistent in effect direction. Utilising elevations in prothrombin fragment F1 + 2 to measure anti-coagulation had a positive association with increased filter life [72].

Fresh Frozen Plasma (FFP) administration was associated with a non-significant increase in the failure rate however this was only reported in one study [53].

Of interest were studies [32, 33, 35, 67, 73] reporting heparin resistance from low anti-thrombin III levels (AT-III). Higher AT-III levels trended to longer filter life, reaching significance in one study [33] while correction of AT-III deficiency was associated with a pooled 18.8% increase in filter life.

The presence of platelet-factor 4 antibodies (PF4) against heparin was significant in one study [74] which went on to demonstrate filter life comparable to non-PF4 positive patients after switching from heparin to danaparoid.

Higher fibrinogen levels were inconsistent in association with filter life with no effect reported by Stefanidis et al. [73] however a 50.8% increase in the failure rate for each 1 g/L increase in fibrinogen reported by Fu et al. [41] (*p* < 0.005).

### Mobilisation and position (Fig. 14)

A single study by Wang et al. [36] challenged the widely held belief that CRRT should be stopped for physiotherapy however the small numbers and trial design is open to observation bias. Another recent study by Toonstra et al. [75] affirmed that patient safety with CRRT and mobilisation can be maintained with care.

Kim et al. [37] explored the fraction of time patients spent in various positions with femoral vascular access and did not show a favoured side however that the supine position was used in nearly 50% of observations. Unfortunately, they did not report the interaction between position and left or right femoral catheters.

## Discussion

This systematic review identified all published studies presenting comparisons of non- anticoagulant factors in relation to filter life. Overall filter life amongst included studies was less than 24 h with wide variation and well short of the multiple days that many filters can last. An interpretative summary of findings is presented in Table 5.

**Table 5:** Summary of findings table

Outcomes	Impact	№ of participants (studies)	Quality of the evidence (GRADE)
Vascular Access interventions to prolong filter life in CRRT			
Temporary Vascular Access Site	Optimal vascular access site ranked by association with longer filter life is: tunneled semi-permanent, femoral or internal jugular, subclavian site.^a^	(9 observational studies)	VERY LOW ^a,b,c^
Tunneled Semi-permanent Catheters vs Temporary Catheters	Tunneled semi-permanent vascular access devices were consistently associated with longer filter life. A significant confounder is that these devices were often larger internal diameter than temporary devices however on the basis of current literature they should be considered in any cases expected to have prolonged CRRT requirement	(4 observational studies)	LOW ^b,c^
Side of Vascular Access Catheter	Overall there is insufficient data and possibility of significant confounding by order of catheter choice such that optimal side of vascular access cannot be determined	(3 observational studies)	LOW ^a^
Catheter length at thoracic vein sites	Favours longer catheter length with atrial placement when thoracic veins utilised. Single study only however unlike most filter life studies this was randomized. No increased in arrhythmias with longer catheter length however underpowered to detect complications.	(1 RCT)	MODERATE ^d^
Catheter lumen size	Only one small study directly measured filter life with catheter size however indirect measures (increased renal dose) in RENAL study supports catheter size as important. Possible benefit from tunneled access may be due to catheter size	(3 observational studies)	VERY LOW ^a,c,e^
Number of vascular access related alarms	Number of vascular access alarms is likely a significant contributor to poor filter life however data is limited	(1 observational study)	VERY LOW ^g^
Access Catheter Type	No significant difference between brands of catheters though trend existed. Tunneled catheters were superior to temporary catheters	(4 observational studies)	LOW ^b,e,g^
Circuit Management Interventions to Prolong Filter Life in CRRT			
Haemofilter Membrane Characteristics	Hollow fibre membranes appear superior to flat plate membranes. It is unclear if an advantage exists for polyacrylonitrile membranes compared to polysulfone or cellulose membranes in regards to filter life. Membrane area was not associated with increased filter life in a single study.	(8 observational studies)	VERY LOW ^h^
CRRT Modality	CVVH is associated with worse filter life in published studies	(7 observational studies)	LOW ^i^
Pre vs Post Dilution in CVVH	One small RCT favoured pre-dilution. Overall affect from all studies trended toward pre-dilution but did not reach significance.	(4 observational studies)	VERY LOW ^i^
Blood Flow Rate	Majority of trials suggest a higher blood flow rate increases filter life however it is unclear over what range this applies. Studies directly comparing low and high blood flow are required.	(9 observational studies)	VERY LOW ^i^
Saline Flushes to Blood Path	There is no evidence to support intermittent saline flushing of the circuit to prolong filter life	(4 observational studies)	VERY LOW ^i^
Education and Alarm Management	Limited evidence suggests focused training to recognise and respond to filter warnings prolongs filter life	(4 observational studies)	VERY LOW ^i^
Patient Factors associated with prolong filter life in CRRT			
Factors with a positive association with filter life	Increasing age (NS), Presence of vaso-active drugs (NS) ^f^, lower pH (sig), Higher APTT (sig), Higher ATIII level (NS), Correction of ATIII deficiency (sig), Mobilization (sig) ^f^, Number of position changes (sig) ^f^		VERY LOW ^b,j,c^
Factors with a negative association with filter life	Being male (NS), Mechanical Ventilation (sig) ^f^, Increased temperature (NS) ^f^, Liver failure with bilirubin > 3 mg/dL (NS) ^f^, Presence of Sepsis (NS) ^f^, Higher SOFA score (sig) ^f^, Higher LOD score (sig) ^f^, Unit increase in ionized calcium (sig), Higher platelet count (sig), RBC transfusion (sig) ^f^, Platelet transfusion (NS) ^f^, FFP transfusion (NS) ^f^, PF4 antibodies ^f^, Elevated fibrinogen (sig) ^f^, Prothrombin fragment (F1 + 2) elevation ^f^		VERY LOW ^b,j,c^
GRADE Working Group grades of evidence: High quality: We are very confident that the true effect lies close to that of the estimate of the effect Moderate quality: We are moderately confident in the effect estimate: The true effect is likely to be close to the estimate of the effect, but there is a possibility that it is substantially different Low quality: Our confidence in the effect estimate is limited: The true effect may be substantially different from the estimate of the effect Very low quality: We have very little confidence in the effect estimate: The true effect is likely to be substantially different from the estimate of effect			

^a^Significant heterogeneity exists and potential for confounders

^b^Timing of catheters during admission has not been studied. Other factors such as choice of catheter length, insertion technique/operator experience at different sites and catheter size at different sites may bias results

^c^Heterogeneity across small observational studies

^d^Unblinded (however unavoidable) however unlikely to affect results

^e^One direct study, strong suggestion that the benefit of tunneled access could be due to catheter size, large RCT post hoc suggests larger size important

^f^Single study, low numbers

^g^Before - after studies with significant risk of other practice changes

^h^Mixed composite of varying quality and study designs with no direct comparison between groups

^i^Some studies used composite interventions

^j^Mostly small observational studies with high risk of bias

### Vascular access

Insertion of vascular access devices for CRRT is a very common occurrence however data to guide optimal catheter site is not strong. The heterogeneity in filter life between femoral and internal jugular siting suggest that unstudied factors such as timing of device in relation to severity of illness, patient factors (such as siting the device in a femoral location for sedation patients or upper body for upright patients) and operator experience may be important. Studies using time to catheter dysfunction [28, 76] as the outcome have similarly mixed results though the only randomised study found no difference [28] between femoral and jugular. Further analysis of large datasets may provide clarity – in particular studies are required that report the order of catheters.

Of interest was the result from Wang et al. [36] that patient mobilisation was not associated with any difference in filter life between femoral and non-femoral catheter sites. CRRT practice has typically minimised patient mobilisation but it could be safe and feasible without therapy interruption [36, 75]. More studies are needed to guide optimal siting of devices in recovering patients in order to allow ambulation while undergoing CRRT.

The randomised study by Morgan [26] demonstrating superiority of atrial over superior vena cava placement may suggest that studies where the femoral site was found superior could have found less difference if thoracic catheter location had been accounted for. Similarly, the advantage of tunnelled devices may result from the more frequent use of live x-ray positioning to ensure optimal placement during insertion as opposed to only follow up x-rays for tip placement for placement within intensive care units.

Offsetting any advantage to filter life of femoral or jugular catheter placement over subclavian access is the possibility of a lower infection risk at subclavian sites [77–79]. However subclavian access is associated with an increased risk of strictures with one study reporting this event in 50% of subclavian veins from short term temporary dialysis catheter placement [80]. Our conclusion is that subclavian placement should remain the last choice of site.

Infection rates rise most per catheter day for femoral and jugular sites [77, 79] thus we suggest that in patients where CRRT is anticipated to extend beyond 7–10 days a tunnelled semi-permanent device via an internal jugular vein could offer the optimal filter life with minimal infection risk.

Femoral sites may also predispose to an increased risk of deep venous thrombosis though this finding is not universal [81, 82]. The clinical risk of lower limb deep vein thrombosis however is greater than upper limb thrombosis.

Variations in catheter design have been extensively described [1–3, 83]. This meta-analysis does suggest a trend toward some catheter designs being superior however to date studies have been small and at high risk of confounding and bias. Future catheter technology ideally should be subject to more rigorous comparisons.

### Circuit factors

The evidence base for justifying decisions regarding optimal CRRT mode of therapy is weak though CVVHD-F does appear to offer superior filter life to CVVH consistently in all studies. Even this conclusion is complicated by multiple interacting factors including anticoagulation choice, blood flow determination, nursing expertise and vascular access.

The literature remains unclear in regards to optimal choice of haemofilter membrane despite several evolutions of this technology. Recent advances such as heparin bonded surfaces to minimise cytokine activation and activation of clotting do not have a strong evidence base to demonstrate superiority in regards to filter life though as individual study authors point out, any effect may be synergistic with choice of anticoagulation [43]. Advantages of improved biocompatibility may not be evident in filter life but in overall patient tolerance with an endotoxic shock model in dogs suggesting improved haemodynamic function with polyacrylonitrile over polysulfone [84] membranes.

Factors such as utilising larger surface area haemofilters to gain longer filter life require more data to demonstrate if any benefit exists from either a filter life, cost or performance perspective. Larger area membranes increase clearance of solutes and for the same flow rates facilitate more rapid restoration of physiologic electrolytes however patient outcome studies are required to determine if this translates to clinical improvement. Conversely there seems little evidence to support saline flushes of circuits used with or without anticoagulation and theoretically this practice may expose the blood path to increased risk of microbial contamination.

There is scope to further investigate optimal blood flow rate in larger datasets or randomised studies while controlling for consistency in anticoagulation and vascular access as this is a simple parameter to adjust that affects both membrane performance and, our results suggest, filter life. Such studies should also address alarm frequency as blood flow increases as we hypothesise that any benefit would reach a maximum after which the frequency of access pressure alarms would increase.

Only two studies were found focussing on change management and strategies for staff managing CRRT despite this therapy consuming significant human resources with frequent bag changes, alarms and poor filter life predominating. Approach to alarms, catheter positioning, choice of blood flow and general trouble shooting likely varies widely yet has been only touched upon sparingly in filter life studies.

### Patient factors

Patient factors associated with filter life are summarised in Table 5 and in general result from a weak evidence base. Many factors that positively influenced filter life are biologically feasible though the trend in a single study of mobilisation improving filter life requires replication.

No studies reported subgroup analysis where indices of body mass index (BMI) were assessed for an effect on filter life. Similarly, no studies described the effects of patient sedation and only one study described an interaction with ventilation despite both factors appearing at the bedside to influence CRRT interruptions.

More work is required in the group with coagulation disorders such as decreased anti-thrombin (heparin resistance), PF4-heparin antibodies and elevated fibrinogen. ‘Clotty’ patients have long been recognised as detrimental to CRRT and though the recent expansion of alternatives to heparin (particularly citrate anticoagulation) have broadened therapeutic options these patients still present a challenge frequently cycling through different strategies empirically or unique un-trialled interventions such as plasma exchange for frequent circuit failure with hyperfibrinogenemia [85].

### Limitations

Future data may clarify if effects such as increasing temperature being negatively associated with filter life remain significant. By not randomising for these outcomes there is a significant risk of unquantified bias explaining the effect or multicollinearity where the observed effect is actually tracking another measured or latent variable [22, 25, 86, 87]. For example, the trend toward shorter filter life with elevated temperature may track with the trend for sepsis and poor filter life. Given that CRRT typically suppresses body temperature in all but the hottest patients this is particularly possible.

Risk of type I error also exists with an example being APTT where the effect size for a unit increase in APTT though statistically significant appears small (and in some studies in an unexpected direction). Higher APTT values would biologically be expected to prolong extracorporeal circuit life however it is an important variable that practitioners of CRRT control or target toward fixed values thus it is not a ‘free’ variable in the regression [86].

The overall quality of evidence from studies is low with few randomised studies and none of significant size. For many variables, effect sizes were extracted from sub-analysis utilising either multiple regression or Cox proportional hazards models and this poses limitations on interpretation. For isolated statistically significant findings from single sub-analysis there is a risk of the effect resulting from pure chance i.e. a type II error – however if an association is biologically plausible and is consistent across several studies then it is a strength of meta-analyses that it won’t be dismissed.

This systematic review highlighted the dearth of randomised studies to guide practice and the overall low quality of most studies. A significant risk of publication bias exists given the 102 anti-coagulant comparison studies in CRRT each of which would have had vascular access, circuit and patient properties that was only presented in sub-analyses of 10 studies – we deemed it impractical to pursue this volume of unpublished data. Given the limitations above the findings of this meta-analysis should be considered as a summary of published data and remain hypothesis generating.

The diversity of current practice and limited numbers of large controlled trials significantly hamper interpretation of findings. However, many effects that reached significance are largely consistent with clinical experience and highlight where clinical decision making and future studies should consider the existing data such as around timing of transfusions, involvement of mobilisation and coagulation strategies in septic patients.

This review is useful in highlighting the weak underpinnings of current clinical practice in this area. Filter life is an objective measurement that can readily be followed within a unit as a quality control and understanding factors that influence variability will aide improvements of this index.

This review also highlighted some omissions in the literature; though studies have looked at operator experience and ultrasound guided insertion in relation to vascular access complications [88–90], filter life has not been assessed as an outcome of ultrasound guided catheter placement. Studies of alarm frequency by catheter site and interactions with patient position need further investigation. No studies reported on an interaction between catheter site and body habitus in regards to filter life – we postulate that patients with centripetal obesity may be more likely to receive jugular catheters and this may interact with filter life. No studies looked at catheter care and locking in relation to filter life.

## Conclusion

Despite the improvements in device technology and usability, filter life remains highly variable across published literature. This is somewhat unsurprising given the myriad of interacting patient, vascular access site and type and circuit factors. Perhaps more surprising is the absence of strong guiding evidence outside of anti-coagulation strategies after over 20 years of therapy delivery. Significant ongoing data collection is required to elucidate the optimal technological and management strategies to enhance current delivery of care to provide optimal performance with minimal disruption at the least cost.

## Abbreviations

AKI: Acute Kidney Injury
CRRT: Continuous Renal Replacement Therapy
CVVH: Continuous Veno-venous Haemofiltration
CVVHD: Continuous Veno-venous Haemodialysis
CVVHD-F: Continuous Veno-venous Haemodiafiltration
Qb: Blood flow (ml/min)
RCT: Randomised Controlled Trial
